# Examining the role of socioeconomic adversity on depressive symptoms during pregnancy among non-Hispanic Black women

**DOI:** 10.1016/j.ssmmh.2025.100463

**Published:** 2025-05-13

**Authors:** Anneliese Long, Amanda L. Thompson, Margaret Bentley, Harlyn G. Skinner, Alexis Woods Barr, Khristopher Nicholas, Ivonne Headley, Heather Wasser

**Affiliations:** a Department of Anthropology, University of North Carolina, Chapel Hill, NC, USA; b Carolina Population Center, University of North Carolina, Chapel Hill, NC, USA; c Department of Nutrition, Gillings School of Global Public Health, University of North Carolina, Chapel Hill, NC, USA; d Center for Health Promotion and Disease Prevention, University of North Carolina, Chapel Hill, NC, USA; e Department of Health Behavior, Gillings School of Global Public Health, University of North Carolina, Chapel Hill, NC, USA; f Harvard T.H. Chan School of Public Health, Harvard University, Boston, MA, USA

## 1. Introduction

There is an increasing need for mental health care among the greater population of the United States, a need which is patterned by preexisting social, demographic and economic injustices. The experience of depressive episodes occurring over the course of pregnancy follows these patterns, and yet remains an understudied area of research. Despite this, the experience of depression is estimated to affect at least between 10 % and 20 % of pregnant people in the United States, with higher estimates for people of color and those living in poverty (Le Strat et al., 2011; Mukherjee et al., 2016; Van Niel and Payne, 2020). Depression represents a collection of symptoms, including hopelessness, despair, empty moods, irritation, lack of concentration, loss of appetite, sleep disturbances, and suicidal ideation (Jahan et al., 2021). These symptoms often lead to a negative impact on an individual’s ability to perform day-to-day activities and functions. Symptoms common with depression, such as sleep and energy disturbances, may be attributed to pregnancy alone, leading to underdiagnosis during this part of the life course (Marcus, 2009). For birthing parents, prenatal depression has been associated with inadequate weight gain, under-utilization of prenatal care, and increased substance use (Marcus, 2009). There is additional evidence that depression during pregnancy increases the risk for the child, with its association to preterm birth, growth restriction, and low head circumference (Ghimire et al., 2021; Li et al., 2020; Marcus, 2009; Van Niel and Payne, 2020). Untreated depression may also increase the risk of impaired social, cognitive, and emotional development of the child (Van Niel and Payne, 2020). Studying depressive symptoms during pregnancy is crucial for the well-being of the birthing parents, particularly among marginalized groups facing multiple, intersecting structural barriers to care (Goodman, 2020; Gotlib et al., 2020; Martin et al., 2024).

The prevalence of prenatal depression varies across sociodemographic groups by socioeconomic status, rurality, and race/ethnicity (Mukherjee et al., 2016; Nidey et al., 2020; Woody et al., 2017). Depressive symptoms are generally understood to be part of a broader landscape of psychosocial stress and overall mental health, with psychosocial stress during pregnancy correlating to significantly higher levels of depression during the perinatal period (Van Niel and Payne, 2020). Known factors that increase the risk of depressive symptoms during and prior to pregnancy include low social support, unplanned or unwanted pregnancy, poor relationship quality, interpersonal abuse, and low socioeconomic status (Brown et al., 2023; Dagher et al., 2021; Martin et al., 2024). Prenatal depression has also been implicated as a significant risk for postpartum depression (Dagher et al., 2021).Critically, the racialized class system within the United States exists upstream of socioeconomic factors, shaping individuals exposure to resources and stressors that can contribute to psychological well-being (Brown et al., 2023). The intersecting experiences of class, race, and gender can greatly impact exposure to stressors, leading to the coalescence of stressors for those that share interconnecting marginalized identities during the sensitive period of pregnancy in which the symptoms may manifest (Brown et al., 2023).

Those below the federal poverty line experience significantly higher rates of depression than those that are not (Benatar et al., 2020; Lefmann and Combs-Orme, 2014). Poverty, the lived experience of unequal distribution of resources, imposes a distinct form of stress operating through the hypothalamic–pituitary–adrenal axis (HPA axis), considered the primary neuroendocrine system orchestrating the stress response (Brisson et al., 2020; Lefmann and Combs-Orme, 2014). Emerging understandings of the biological effects of poverty highlight the role of precarity and uncertainty in the experience of stress related to resource insecurity, such as food and housing insecurity associated with poverty (Hoke and Long, 2023). A number of barriers may exist for vulnerable communities and individuals to access mental health care, particularly people from racial and ethnic minority groups, including lack of mental health providers, lack of effective prenatal behavioral health systems, and stigma that collectively and independently limit access to care (Gennaro et al., 2020). Systemic racism is a key determinant of class, given that the class system in the United States historically has been built to uphold the power of white individuals, which has led to the disproportion of communities and people from racial and ethnic minority groups experiencing poverty, along with interpersonal and structural discrimination (Geronimus et al., 2006; Gutin and Hummer, 2021; Mukherjee et al., 2016).

Socioeconomic status is defined as the social status associated with an individual or group, often measured through a combination of education, income and occupation (Braveman et al., 2001; Cirino et al., 2002; Darin-Mattsson et al., 2017; Demakakos et al., 2008). Individual characteristics including education level, income, home ownership, and parental education have been used previously to measure socioeconomic position (Braveman et al., 2001; Cirino et al., 2002; Darin-Mattsson et al., 2017). Increasingly, community-level measures that center ‘place’ in the measure of social status have been leveraged to examine their role of health outcomes (Arcaya et al., 2016; Kind et al., 2014; Yang and Gustafsson, 2004). Socioeconomic measures at the community level represent patterns of social organization, structure, stratification, or environment (Arcaya et al., 2015, 2016; Moss et al., 2021). People are fundamentally and relationally embedded into places while simultaneously maintaining and producing the places they and other individuals encounter (Crimmins et al., 2014). Therefore, community socioeconomic status is thought to capture elements of deprivation, economic inequality, availability of resources, and structures of economic opportunity (Arcaya et al., 2016). Place-based measures of status offer a unique and complementary addition to individual measures of socioeconomic status, though the relationship between these components is not yet well understood.

Despite the need for intersectional approaches to the study of depressive symptoms during pregnancy, research is limited on the concurrent experience of sociodemographic and geographic factors during the perinatal period. There is especially a need to explore these intersections among non-Hispanic Black pregnant people, who experience a higher risk of exposure to discrimination coupled within neighborhood structures (English et al., 2014; Ertel et al., 2012; Giurgescu et al., 2017; Giurgescu et al., 2015; Giurgescu et al., 2015; Walker et al., 2024). The objective of the present analysis to fill this gap by examining the role of both individual measures and neighborhood measures of social status among a sample of Black women and pregnant individuals in North Carolina on the outcome of depressive symptoms during the pregnancy period. It is hypothesized that those experiencing a higher burden of neighborhood disadvantage or deprivation, and lower measures of individual SES, would exhibit the highest odds of depressive symptoms during pregnancy.

## 2. Materials and methods

Using data from the Mothers and Others study, we used individual-level socio-demographic data and residential addresses during pregnancy to examine the role of social status and place on depressive symptoms. We utilized area deprivation index (ADI) and the disadvantage index developed by the National Neighborhood Data Archive (NaNDA) which have been widely used as measures of socioeconomic adversity at a community-level (Kind et al., 2014; Kind and Buckingham, 2022; Melendez et al., 2022; Singh, 2003). The Area Deprivation Index (ADI) was developed by Singh and colleagues to measure a multidimensional characterization of an area’s socioeconomic position using census block groups on the national level and state level based on American Community Survey (ACS) data, collected every five-years by the U.S. Census Bureau (Singh, 2003). Area-level indices may capture distinct components and experiences of neighborhood level socioeconomic status (Arcaya et al., 2015; Kind and Buckingham, 2022; Singh, 2003). These may impose or mitigate distinct stressors that affect the risk of depression, particularly during periods of transition and change that may already involve an increased level of stress, such as during pregnancy (Bennett et al., 2004; Ghimire et al., 2021; Mukherjee et al., 2016).

### Study Design.

The objective of the Mothers and Others study was to assess the efficacy of a family-centered and multi-component intervention, among Non-Hispanic Black families, which represent a minority population with a higher risk for childhood obesity (Wasser et al., 2017, 2020). The study design was a randomized controlled trial of 430 pregnant participants, and each study participant selected one study partner (i.e. other) who participated in the duration of their study enrollment. At baseline, mothers identified the study partner by answering the question, “Who is the person, other than a doctor or healthcare professional, that is most important to your decision-making about infant care or that will be involved in caring for the infant during the first few months after his/her birth?” (Wasser et al., 2020). In the intervention group, participants and their study partners received pre-emptive information on breastfeeding, responsive feeding, and non-food infant soothing techniques, as well as content on complementary feeding practices, infant sleep, and TV/media. Participants randomized into the control group received information on child safety and identified one “other,” who only completed study assessments. In both groups, intervention content was delivered through home visits by trained peer educators and newsletters. The Mothers and Others study took place from 2013 to 2017 in central North Carolina (Wasser et al., 2017). Pregnant people who were planning to deliver at three local hospitals in central North Carolina were primarily recruited by trained recruitment specialists in prenatal clinics, alongside flyers posted in community-based locations (e.g. churches, libraries) and announcements to parenting listservices. The eligibility criteria at baseline for participants included pregnant people and women who self-identified as non-Hispanic Black and were ages 18–39 years with a singleton pregnancy, spoke English, were less than 28 weeks gestation, were planning to stay in the area, and could identify one study partner. Data on depressive symptoms were collected at 28 weeks gestation, prior to randomization procedures, which was the baseline assessment for participants following their study enrollment (Wasser et al., 2017). Depressive symptoms were self-reported and measured by the Center for Epidemiological Studies (CES-D) instrument (Lewinsohn et al., 1997; Radloff, 1977; Shaffer, 2014). This study protocol was reviewed and approved by the institutional review board of the University of North Carolina at Chapel Hill [Trial Registration: NCT01938118].

### Study Sample.

**Fig. 1.**
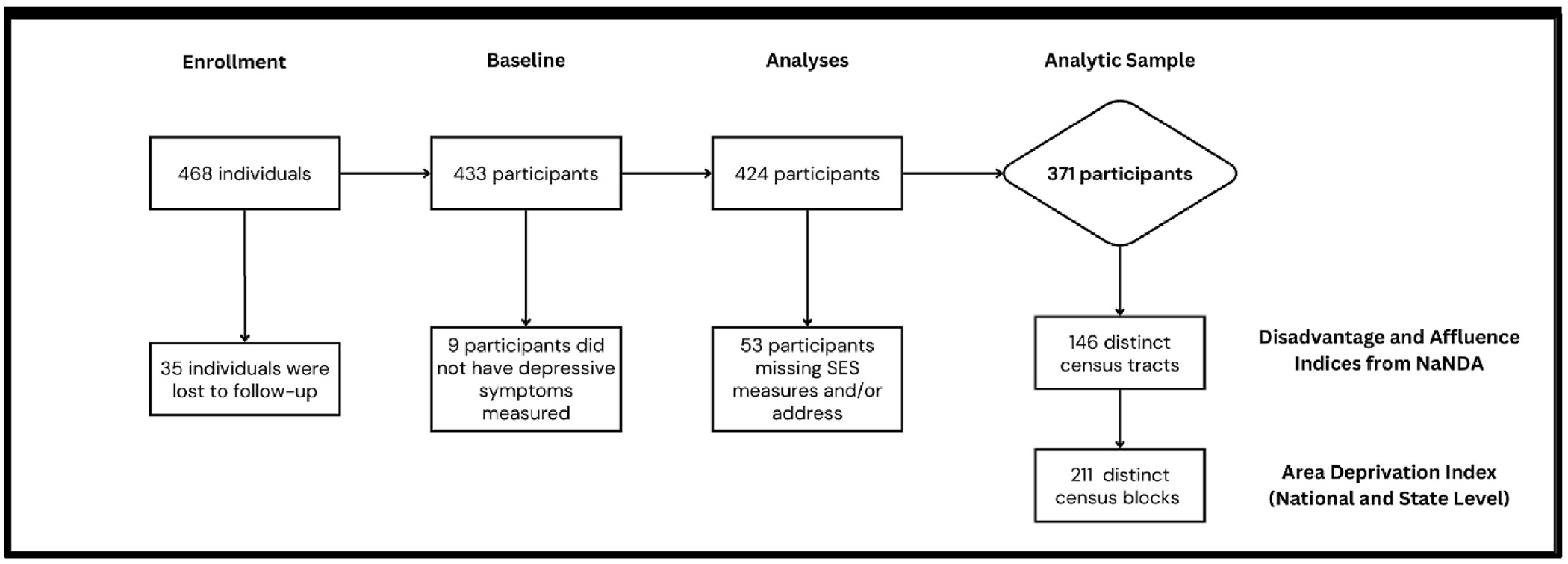
Mothers and Others Participants and Number of Geographic Units included in this analysis.

There were a total of 430 participants enrolled in the study with one participant having incomplete baseline data and therefore not randomized (Wasser et al., 2017). For this analysis, participants that were missing depressive symptoms measurement (N = 9), socioeconomic status (SES) variables used to generate the composite score, described in detail below, (N = 2), or address data (N = 51), were excluded. The total analytic sample for analysis was 371 participants. A detailed flow chart of participants included in this analysis is included in Fig. 1.

### Composite Individual Socioeconomic Status (SES) Measure.

Individual socioeconomic status was measured using a composite metric developed for the purpose of this analysis based on existing literature of SES measures (Braveman et al., 2001; Cirino et al., 2002; Darin-Mattsson et al., 2017). For each participant, the metric summed values (0 = No and 1 = Yes)for food insecurity status, income below 185th of the federal poverty line based on household size (0/1), and less than college education level (0/1). The composite also included measurements for the use and eligibility (0 = Not Used/Eligible, 1 = Used/Eligible, 2 = Not Used/Eligible) for the following social service programs: Women, infants, and children (WIC), Supplement Nutrition Assistance Program (SNAP) (0/1/2), and Medicare (0/1/2). It was reasoned that individuals eligible for social support services but not utilizing them, regardless of the cause, would be most vulnerable and experience the highest level of resource deprivation compared to those who were either using these services or ineligible for them. Use of these social support programs were incorporated into the composite score additively to the food security status, income, and education binary values. In total, the additive composite SES score represented numeric values from 0 to 8, with 8 indicating the lowest level of SES and 0 representing the highest level of SES. While SES was analyzed continuously in all logistic regression models. For descriptive purposes only, presented in Table 1, individual SES was categorized as low (0–2), moderate (3–5), and high (6–8). Each individual component of the composite score was examined to determine its relationship with depressive symptoms, however, the totality of the score is used in this analysis to capture a comprehensive measure of socioeconomic status.

### Area-Level Deprivation (ADI) Index.

An in-depth description of the methodologies used to develop the measure has been previously published (Kind et al., 2014; Singh, 2003). In brief, Singh and colleagues developed ADI based on factors identified from 1990 Census and American Community Survey data based on theoretical relevance and previous empirical research. Factor analysis and principal-components analysis on indicator variables were used in index construction. These indicators included educational distribution (percentage of the population with less than 9 years and with 12 or more years of education), median family income, income disparity, occupational composition, unemployment rate, family poverty rate, percentage of the population below 150 % of the poverty rate, single-parent household rate, home ownership rate, median home value, median gross rent, median monthly mortgage, and household crowding (Singh, 2003). The other indicators were percentages of households without access to a telephone, plumbing, or motor vehicles, English language proficiency, percentage of urban population, and percentage of immigrant population (Singh, 2003). The factor score coefficients of the models were used to weigh the 17 indicators comprising the index, with poverty, income, and education having the largest relative weights. At the national level, the scores represent percentiles ranging from 1 to 100 of relative deprivation, with the highest score representing the most deprived areas at the national level (Kind et al., 2014; Singh, 2003, 2003, 2003). At the state level, the scores are also percentiles, however, these range from 1 to 10, with 10 representing the most deprived areas (Kind et al., 2014; Singh, 2003, 2003, 2003). Though in all multivariable models the ADI indices were examined continuously, for descriptive purposes presented in Table 1 and for the purpose of this descriptive analyses of fixed probabilities of high depressive symptoms controlled for covariates, ADI was also categorized as low (1–3), moderate (4–6), and high (7–10). Data were extracted from Neighborhood Atlas^®^ from the Center for Health Disparities Research at the University of Wisconsin School of Medicine and Public Health (Kind et al., 2014; Kind and Buckingham, 2022).

### National Neighborhood Data Archive (NaNDA) Disadvantage Index.

The measure of disadvantage from NaNDA represents the mean of four measures from the American Community Survey at the census tract level, with data derived during the study period of 2013–2017: proportion of female-headed families with children, proportion of households with public assistance income, proportion of people with income in the past 12 months below the federal poverty level, and the proportion of people in the civilian labor force above the age of 16 years old that were unemployed (Melendez et al., 2022). The construction of the index variables has been published previously, and used principal factor analysis with an orthogonal varimax rotation of ten census indicators, aimed at creating an index containing a set of factors that capture the spectrum of neighborhood characteristics (Morenoff et al., 2007). The results indicated three separate factors, which the authors identified as neighborhood disadvantage, neighborhood affluence, and neighborhood ethnicity and immigration distribution (Melendez et al., 2022; Morenoff et al., 2007). The purpose of the present study included use of the neighborhood disadvantage factor index, which was multiplied by 100 to transform the value to a percentage of the population.

### Covariates.

The covariates identified and used in this analysis include participant age, perceived social support (PSS) from family, perceived social support (PSS) from friends, partnership status, and number of children in the household. These variables were selected based on existing empirical evidence on antenatal depression (Bedaso et al., 2021; Gentile, 2017; Mukherjee et al., 2016; Staneva et al., 2015; Wasser et al., 2017).

#### 2.1. Statistical analysis

We descriptively analyzed the distribution of the following characteristics, which were all assessed at baseline. We categorized age as a continuous variable in five groups: less than 20 years old, between 20 and 24 years old, between 25 and 29 years old, between 30 and 34 years old, and older than 34 years old. Partnership status was categorized as a binary variable whether the participants reported being married or in a domestic partnership versus those that did not report a partner. A binary income variable was created based on household income above or below the 185th percentile of the federal poverty line. Education was categorized at three levels: less than high school, high school or high equivalent, and some college or greater. For WIC, SNAP, and Medicaid, we examined each at three levels: those eligible and enrolled, those eligible and not enrolled, and those not eligible and not enrolled. Food security status was assessed using the USDA Food Security Scale and was categorized at three levels: very low food security, low/marginal food security, and high food security (Economic Research Service, 2012) State-level and National-level ADI percentiles were also categorized based on the following cut points based on the trimodal distribution of the continuous variable: the categories for state-level ADI were low if equal to or between 1 and 3, moderate if equal to or between 4 and 7, and high if equal to or between 9 and 10. The categories for national-level ADI were low if less than or equal to 34, moderate if equal to or between 35 and 67, and high if equal to or between 68 and 100. Descriptive characteristics are summarized in Table 1.

A total of ten adjusted regression models were used to examine the effect of individual socioeconomic status (SES), neighborhood socioeconomic status measures, and depressive symptoms. Individual level socioeconomic status, State-level ADI and National-level ADI were initially examined independently of one another in models 1, 2, and 3. In models 4 and 5, individual level SES and State-level ADI, and individual level SES and National-level ADI, were examined within the same model, respectively. In models 6 and 7, interaction terms for individual SES and National-level ADI, then individual SES and state-level ADI. In models 8, and 9, we used the disadvantage variable derived from the disadvantage score to examine its independent effect on depressive symptoms, as well as its effect on depressive symptoms with individual SES included in the model. These interaction terms assess the extent to the association of depressive symptoms and individual SES differ by regional/state deprivation status. In model 10, we used an interaction term between individual level SES and the disadvantage score to examine their joint effect on depressive symptoms. All models are presented in Table 2.

**Fig. 2.**
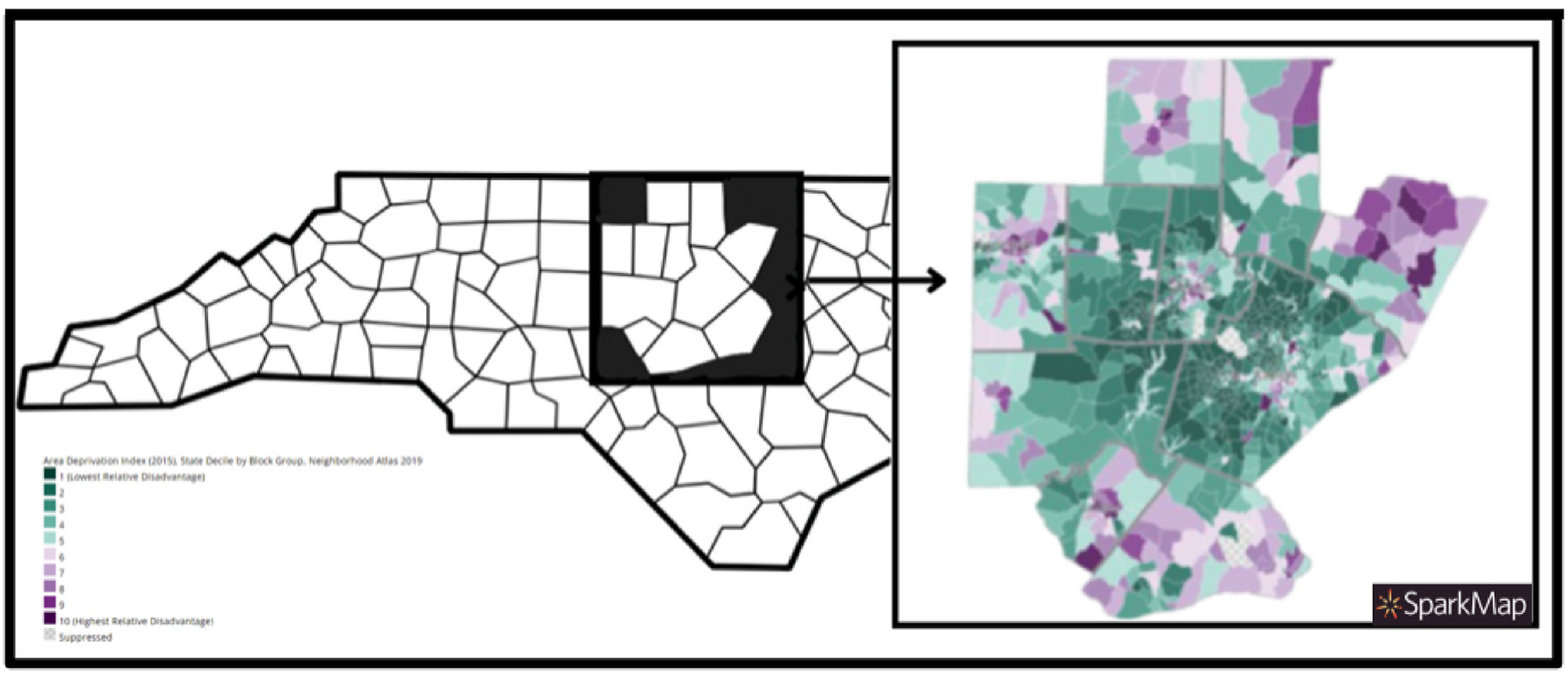
State area deprivation index (ADI) distribution for participant Counties by census block group in North Carolina.

**Fig. 3.**
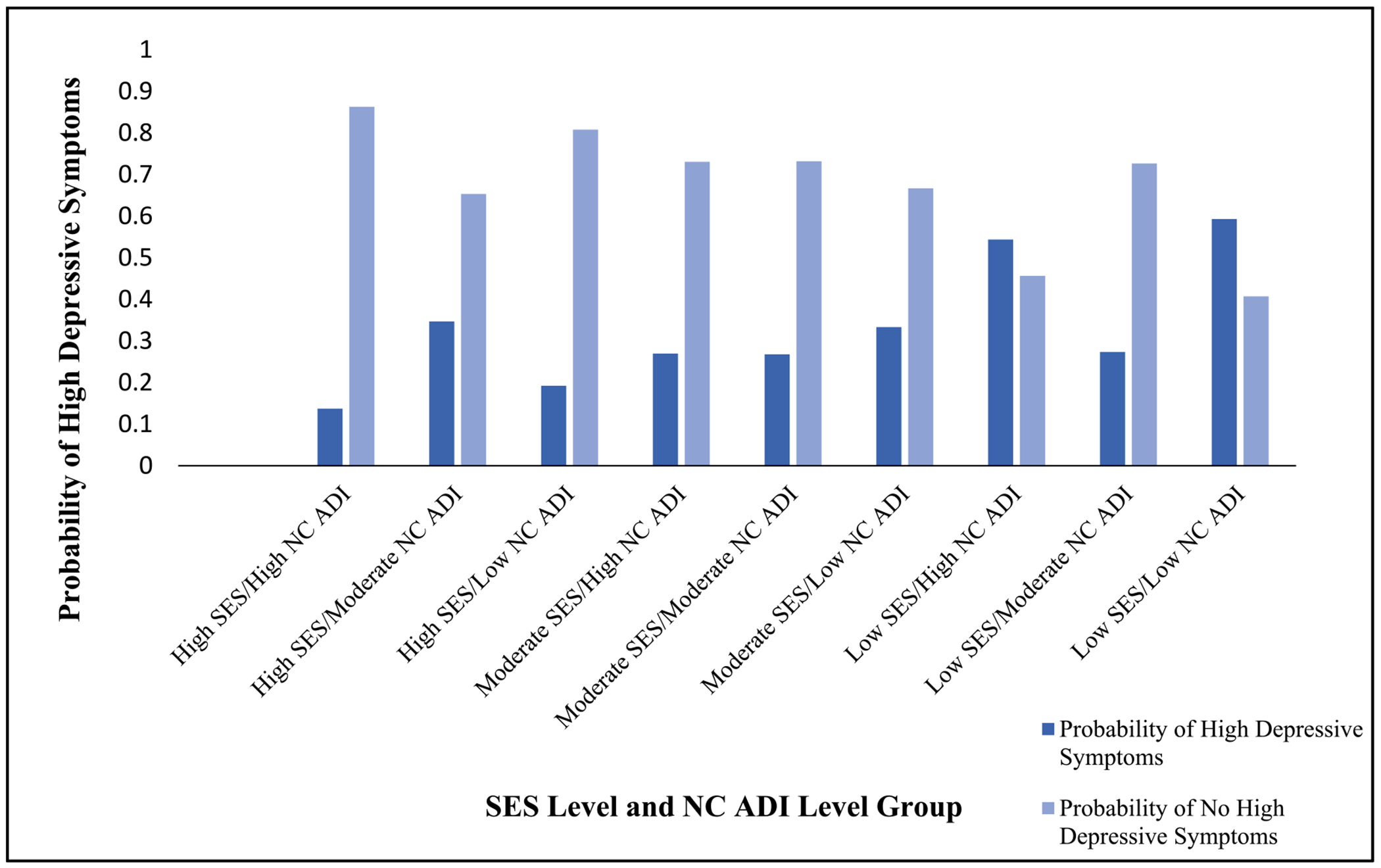
Adjusted Predicted Probability of High Depressive Symptoms by Individual Socioeconomic Status (SES) and North Carolina Area Deprivation (ADI) Group^a, b^ ^a^Predicted probabilities are adjusted for maternal age, perceived social support from family, perceived social support from friends, number of children, and partnership status. ^b^For ease of interpretation, we plotted the interaction term for individual socioeconomic status and North Carolina Area Deprivation in tertiles. SES: Socioeconomic Status Composite Measure; ADI: Area Deprivation Index.

Fig. 2 illustrates the distribution of state-level ADI by census block for each county where participants resided (Kind et al., 2014; Kind and Buckingham, 2022; University of Wisconsin School of Medicine and Public Health). Fig. 3 illustrates predicted probabilities were descriptively examined for the interaction between North Carolina ADI and individual level SES using the margins function, stratified by each combination of SES and NC ADI.

## 3. Results

### Descriptive characteristics.

Most study participants were between the ages of 20 and 29 years old (67 %), were not married or in a domestic partnership (62 %), had previous children (58 %), had low income (73 %), and had an education-level of some college or greater (52 %) (Table 1). All study participants self-reported their race/ethnicity as either Non-Hispanic Black or Non-Hispanic African American. There were few participants that were not enrolled in social support programs, with most enrolled in WIC (88 %), SNAP (69 %), and/or Medicaid (72 %). Most participants reported to be food secure based on the USDA Food Security Scale (78 %). The overall proportion of participants in the total sample (N = 371) exhibiting high levels of depressive symptoms was approximately 32 %. A high-level of depressive symptoms were more frequent among those with low income. Among those with high depressive symptoms, it was higher among those without previous children. Overall, the proportion of participants with depressive symptoms was lower among those who were married or in domestic partnerships, as well as for participants who were older.

### Adjusted logistic regression models.

The relationship between individual SES and depressive symptoms was hypothesized to vary according to neighborhood deprivation strata. Examination of individual SES, State ADI, National ADI, and neighborhood disadvantage, independent of one another, showed significance only for individual SES and maternal depressive symptoms. One unit increase in the individual low SES measure was associated with a 24 % increase in the odds of high depressive symptoms (OR: 1.24; 95 % CI: 1.07, 1.44). Within this relationship, perceived social support from family members and partnership status were both strongly associated with a reduction in risk of depressive symptoms, a 14 % and 45 % respective decrease in odds of maternal depression (OR: 0.86; 95 % CI: 0.79, 0.94 and OR: 0.55; 95 % CI: 0.31, 0.97). Individual SES models adding State ADI, National ADI, or neighborhood disadvantage did not change results; individual SES remained strongly associated with increased odds of high depressive symptoms with 23 % greater odds for all models (OR: 1.23; 95 % CI: 1.06, 1.43). Perceived social support from family members and partnership status remained significantly associated as well with approximately the same effect sizes. Interaction terms were examined to investigate whether the relationship between individual SES and maternal depressive symptoms varied by ADI or neighborhood disadvantage strata. There was no evidence of interaction between individual SES and neighborhood disadvantage or any ADI. These results are presented in Table 2.

### Adjusted logistic regression models, interaction terms.

In examining the relationship between individual SES and each of the ADI values with interaction terms, there was no evidence of an interaction between either index or individual SES. For the interaction model between individual SES and neighborhood disadvantage, there was no evidence of an interactive relationship.

## 4. Discussion

For the adjusted logistic regression results, individual SES was the only exposure that was significantly associated with an increased risk of high depressive symptoms. The relationship between depressive symptoms and the computed individual-level SES score is consistent with previous findings that lower individual SES is associated with a higher risk of depression (Ridley et al., 2020). The stress created by the structural inequities of poverty, such as the lack of resources, uncertainty of housing, food, and other needs are hypothesized to be key reasons for this relationship (Bennett et al., 2004; Hoke and Long, 2023; Mao, 2022; Ridley et al., 2020). The experience of precarity precipitated by structural inequalities of the class system and the racial categorization system of the United States are hypothesized to underlie the decrease in mental well-being, which may have further cascading disruptions to quality of life (Evans et al., 2012; Hoke and Long, 2023). Previous work among pregnant Non-Hispanic Black women has suggested that lifetime trauma exposure may play a role, which has been associated with depressive symptoms, anxiety, and generalized stress (Dailey et al., 2011; Powers et al., 2020). Familial incarceration and perceived discrimination, both of which disproportionally affect Black communities, have also been associated with higher levels of depressive symptoms (Patterson et al., 2021; Qin et al., 2020).

Within all adjusted logistic regression models, perceived social support from family members and partnership status were significantly associated with decreased odds of depressive symptoms. These findings coincide with previous evidence that social support is protective against depressive symptoms among Non-Hispanic Black women (Benca-Bachman et al., 2020; Chatters et al., 2015; Giurgescu et al., 2015; Shim et al., 2012). Based on existing research, there is a clear link between social support, stress, and mental health during pregnancy and postpartum, though it is less clear if there is a causal relationship or mediating pathway between these (Reid and Taylor, 2015). Previous studies have demonstrated that social support may be a mean to manage and address stressors, including the experience of discrimination (Bedaso et al., 2021; Eick et al., 2020; Qin et al., 2020; Reid and Taylor, 2015). Thus, interventions that increase levels of social support during the this important part of the life course may reduce the risk of depression during pregnancy (Bedaso et al., 2021). Partnership status has also been strongly associated with a decreased risk in depressive symptoms during pregnancy, with pregnant individuals that perceive stronger support from their partners reporting lower levels of emotional distress (Reid and Taylor, 2015). The analyses did not find an effect for maternal age, number of children, or perceived social support from friends were not associated with depressive symptoms, though future research should explore these components in-depth to better understand their role on depressive symptoms.

While individual SES was strongly associated with the risk of depressive symptoms, neighborhood deprivation and disadvantage, as measured by State ADI, National ADI, and the neighborhood disadvantage score by the NaNDA, were not associated with the risk of depressive symptoms within this study sample. *Our results are consistent with a systematic review on neighborhood characteristics and depressive symptoms, which has suggested that structural features, such as socioeconomic composition, were less consistently associated with depressive symptoms than with social processes such as perceived disorder, violence and safety* (Ivey et al., 2015; Mair et al., 2008, 2010; Schootman et al., 2007). *For instance, perceived neighborhood environment, measured by questions adapted from the* Neighborhood Physical Environment Scale, *Neighborhood Problems Scale, and the Perceived Neighborhood Scale, among pregnant Non-Hispanic Black women has been associated with depressive symptoms* (Giurgescu et al., 2015). *Neighborhood racial composition measures have been associated with depressive symptoms among Non-Hispanic Black participants, with the percentage of White individuals residing in a neighborhood positively associated with depressive symptoms among Non-Hispanic Black individuals* (English et al., 2014). *Increased exposure to discrimination, which is also positively associated with depressive symptoms, has been hypothesized as an explanatory factor* (English et al., 2014; Qin et al., 2020). Racial residential segregation may also play a distinct and complex role in prenatal depression risk, as some have found that living among higher proportions of same race/ethnicity as protective, while others have demonstrated negative effects of less diverse neighborhoods (Haight et al., 2023; Kelly-Taylor et al., 2024). *Overall*, these results suggest that proximate social environments at the household and individual level, represented by a composite SES measure, are stronger predictors of prenatal depressive symptoms than distal structural environments, measured by three indices of neighborhood deprivation. However, it remains possible that neighborhood and community levels factors that are predictive of depressive symptoms in this population were not represented by these indices.

## 5. Strengths and limitations

A key strength of our study is the comprehensive exploration of socioeconomic-status at the individual level coupled with two distinct community-level markers of disadvantage and deprivation. This study also includes only non-Hispanic Black birthing parents, who are an important population at increased risk for depressive symptoms, likely due to multiple, intersecting barriers to mental well-being, particularly during the period of pregnancy. Nonetheless, there are a few, important limitations to our study. While the ADI and the NaNDA disadvantage index both are considered to measure residential environments and widely utilized to capture it, it is inaccurate to conclusively consider them to measure neighborhoods. A census block or census tract does not truly represent a “neighborhood”, as neighborhoods are fundamentally socially constructed spaces that are unlikely to align with census data (Fowler et al., 2020). Therefore, neighborhood socioeconomic status remains difficult and elusive to measure without the use of in-depth, ethnographic data to determine the boundaries of one’s perceived neighborhood (Flynn and Mathias, 2020; Hwang, 2016; Madrazo, 2019). For our study participants, we were limited to address data only and therefore cannot determine the perceived boundaries of individual’s neighborhoods. Additionally, we do not have measures of participants’ perceptions of neighborhood quality and safety. There is likely to be a social network role beyond the neighborhood level, such as where an individual works, studies, or otherwise may be immersed in other places related to their lives and livelihoods. It is unclear based on current evidence to what extent the primary residence plays compared to other activity spaces; therefore, this is a limitation to this study (Coulton et al., 2013). It also remains possible the factors at the neighborhood level that are predictive of depressive symptoms were not captured by our measures of deprivation and disadvantage. Overall, we maintain that the measurement of community-level deprivation and disadvantage remains a valuable component of the lived experience of poverty, despite limitations in available indicators. Finally, income remains a difficult factor to measure, particularly among vulnerable populations (Parolin, 2019). Income reporting is a common problem in research examining the relationship between income and health outcomes, given that participants may fear judgement or other consequences from accurately reporting their incomes (Proctor et al., 2015). Therefore, we categorized income beyond the 185th percentile of the federal poverty line rather than as individual income brackets, given the likelihood of misclassification. The study design aimed to in part address this limitation through a comprehensive composite score of SES, rather than using income alone. Despite potential limitations, we observed a strong relationship between depressive symptoms and socioeconomic status among our study participants.

## 6. Conclusions

Within this cohort of non-Hispanic Black pregnant women in central North Carolina, lower individual SES was associated with a higher risk of depressive symptoms during pregnancy. The prevalence of depression within the Mothers and Others study was higher than the national average, with approximately 32 % of the overall proportion of participants exhibiting high levels of depressive symptoms, compared to the estimated national average of approximately 13 % during the second trimester of pregnancy (Bennett et al., 2004). Further, both social support from family and partnership status were associated with a decreased risk of depressive symptoms. Neighborhood deprivation and disadvantage, measured by three indices, were not strongly associated with the risk of depressive symptoms. These findings suggest that immediate social environments at the household and individual levels are more powerful predictors of depressive symptoms during pregnancy than broader community factors, as measured by three indices of neighborhood deprivation. The strong association between social support from family and partnership status and depressive symptoms counter individualistic models of depression treatment, providing evidence for the inclusion of community-based models, and perhaps, social network models centered around place.

## Figures and Tables

**Table 1:** Socioeconomic Status, Area Deprivation, and Maternal Depressive Symptoms stratified by participant characteristics.

	Total Sample (N = 371)		Participants Exhibiting Depressive Symptoms^a^ (N = 117)		Participants Not Exhibiting Depressive Symptoms^b^ (N = 254)	
			
	N	%	N	%	N	%
Age, years						
<20	31	8.4	9	7.7	22	8.7
20-24	126	34.0	49	41.9	77	30.3
25-29	123	33.2	38	32.5	85	33.5
30-34	65	17.5	14	12.0	51	20.1
>34	26	7.0	7	6.0	19	7.5
Married/Domestic Partnership						
Yes	112	30.2	22	18.8	90	35.4
No	259	69.8	95	81.2	164	64.6
Previous Children						
Yes	216	58.2	47	40.2	146	57.5
No	155	41.8	70	59.8	108	42.5
Low Income^c^						
Yes	271	73.1	91	77.8	180	70.9
No	100	27.0	26	22.2	74	29.1
Education-Level						
Less than high school	50	13.5	23	19.7	71	16.6
High school or greater	321	86.5	94	80.4	357	83.4
WIC Status						
Eligible/Enrolled	325	87.6	105	89.7	220	86.6
Eligible/Not Enrolled	19	5.1	8	6.8	11	4.3
Not Eligible	27	7.3	4	3.4	23	9.1
SNAP Status						
Eligible/Enrolled	254	68.5	85	72.7	169	66.5
Eligible/Not Enrolled	64	17.3	23	19.7	41	16.1
Not Eligible	53	14.3	9	7.7	44	17.3
Medicaid Status						
Eligible/Enrolled	268	72.2	87	74.4	181	71.3
Eligible/Not Enrolled	60	16.2	20	17.1	40	15.8
Not Eligible	43	11.6	10	8.6	33	13.0
Food Security Status^d^						
Secure	288	77.6	71	39.3	217	85.4
Insecure	83	22.4	46	60.7	37	14.6
State-Level ADI (Census Block)^e^						
Low	130	35.0	40	34.2	90	35.4
Moderate	201	54.2	61	52.1	140	55.1
High	40	10.8	16	13.7	24	9.5
National-Level ADI (Census Block)^f^						
Low	54	14.6	16	13.7	38	15.0
Moderate	218	58.8	61	52.1	157	61.8
High	99	26.7	40	34.2	59	23.2

N: Number; %: Percentage; WIC: Women, Infants, and Children; SNAP: Supplemental Nutrition Assistance Program; ADI: Area Deprivation Index.

a Center for Epidemiologic Studies Depression Scale >16.

b Center for Epidemiologic Studies Depression Scale ≤16.

c Defined as income below the 185th percentile of the federal poverty line, adjusted for household size.

d Based on USDA food security scale.

**Table 2:** Associations between socioeconomic status, neighborhood disadvantage, and maternal depressive symptoms.

Variable	Model 1: SES	State ADI Models			National ADI Models			NaNDA Models		
		Model 2: State ADI	Model 4: SES and State ADI	Model 7: SES and State ADI Interaction	Model 3: National ADI	Model 5: SES and National ADI	Model 6: SES and National ADI Interaction	Model 8: NaNDA Disadvantage	Model 9: SES and NaNDA Disadvantage	Model 10: SES and NaNDA Disadvantage Interaction
									
	OR (95 % CI)	OR (95 % CI)	OR (95 % CI)	OR (95 % CI)	OR (95 % CI)	OR (95 % CI)	OR (95 % CI)	OR (95 % CI)	OR (95 % CI)	OR (95 % CI)
SES	1.24** **(1.07, 1.44)**	–	1.23** **(1.06, 1.43)**	1.37 (0.99, 1.89)	–	1.23** **(1.06, 1.43)**	1.42 (0.94, 2.16)	–	1.23** **(1.06, 1.43)**	1.07 (0.80, 1.43)
State ADI	–	1.06 (0.96, 1.17)	1.05 (0.95, 1.16)	1.18 (0.85, 1.63)	–	–	–	–	–	
SES*State ADI Interaction	–	–	–	0.98 (0.92, 1.04)	–	–	–	–	–	
National ADI	–	–	–	–	1.01 (1.00, 1.02)	1.01 (1.00, 1.02)	1.02 (0.98, 1.06)	–	–	
SES*National ADI Interaction	–	–	–	–	–	–	1.00 (0.99, 1.00)	–	–	
NaNDA	–	–	–	–	–	–	–	1.02 (0.99, 1.05)	1.01 (0.99, 1.04)	0.96 (0.87, 1.07)
SES*NaNDA Interaction	–	–	–	–	–	–	–	–		1.01 (0.99, 1.03)
Maternal Age	1.00 (0.95, 1.04)	0.99 (0.94, 1.03)	1.00 (0.95, 1.05)	1.00 (0.95, 1.05)	0.99 (0.94, 1.03)	1.00 (0.95, 1.05)	1.00 (0.95, 1.05)	0.99 (0.94, 1.04)	1.00 (0.95, 1.05)	1.00 (0.95, 1.05)
Perceived Social Support – Friends	1.00 (0.96, 1.04)	1.00 (0.96, 1.04)	1.00 (0.96, 1.04)	1.00 (0.96, 1.04)	1.00 (0.94, 1.03)	1.00 (0.96, 1.04)	1.00 (0.96, 1.04)	1.00 (0.96, 1.04)	1.00 (0.96, 1.05)	1.00 (0.96, 1.04)
Perceived Social Support – Family	0.86*** **(0.79, 0.94)**	0.87*** **(0.80, 0.94)**	**0.86** *** **(0.79, 0.94)**	0.86*** **(0.79, 0.94)**	0.86*** **(0.80, 0.94)**	0.86*** **(0.79, 0.94)**	0.86*** **(0.79, 0.94)**	0.87*** **(0.80, 0.94)**	0.86*** **(0.79, 0.94)**	0.87*** **(0.80, 0.94)**
Partnership Status	0.55* **(0.31, 0.97)**	0.50* **(0.29, 0.88)**	0.55* **(0.31, 0.98)**	0.55* **(0.31, 0.97)**	0.50* **(0.28, 0.87)**	0.55* **(0.31, 0.97)**	0.54* **(0.31, 0.96)**	0.50* **(0.28, 0.88)**	0.55* **(0.31, 0.97)**	0.54* **(0.30, 0.96)**
Number of Children	0.99 (0.83, 1.18)	1.03 (0.87, 1.23)	0.99 (0.83, 1.18)	0.99 (0.82, 1.18)	1.03 (0,87, 1.23)	**0.99 (0.31, 0.97)**	0.99 (0.82, 1.18)	1.02 (0.86, 1.22)	0.98 (0.82, 1.17)	0.99 (0.82, 1.18)

OR: Odds Ratio; 95 % CI: 95 % Confidence Interval; SES: Socioeconomic Status Composite Score; ADI: Area Deprivation Index; NaNDA: National Neighborhood Data Archive.

*** p < 0.001,

** p < 0.01,

* p < 0.05.
